# Research on plant characteristics and application strategies for the planning and design of herbal gardens

**DOI:** 10.3389/fpls.2025.1688409

**Published:** 2025-11-26

**Authors:** Xiaohua Lin, Zhaocheng Wu, Junmin Liu, Liwen Gao, Ruoting Zhan

**Affiliations:** 1 School of Pharmaceutical Science, Guangzhou University of Chinese Medicine, Guangzhou, Guangdong, China; 2 Guangdong Agribusiness Tropical Agriculture Institute Co., Ltd, Guangzhou, Guangdong, China; 3 School of Medical Information Engineering, Guangzhou University of Chinese Medicine, Guangzhou, Guangdong, China; 4 Human Resource Department, Guangzhou University of Chinese Medicine, Guangzhou, Guangdong, China

**Keywords:** herbal garden, planning and design, plant characteristics, statistics, application strategies

## Abstract

Herbal gardens have a long history worldwide and serve important functions in education, science popularization, cultural dissemination, tourism, and convalescence. In recent years, growing interest in health, nature, and culture has led numerous institutions to establish herbal gardens of various scales. However, standardized research on the planning and design of herbal gardens is still lacking, especially regarding species selection, planting layout, and the exploration and functional utilization of medicinal plant properties. This study conducted a detailed analysis of over 1,000 medicinal plant species in the Herbal Garden of Guangzhou University of Chinese Medicine, focusing on taxonomy, plant habit types, ecological habits, aromatic and ornamental characteristics, medicinal functions, edible properties, and cultural significance. Based on this analysis, the study proposed design principles rooted in ecological diversity, health and safety, and practical functionality, and proposed context-specific, characteristic-driven garden planning to maximize the educational, therapeutic, and public outreach potential of herbal gardens. The findings aim to provide theoretical and practical guidance for the planning and design of diverse types of herbal gardens.

## 1 Introduction

There are approximately 500,000 species of plants, fungi, cyanobacteria, and brown algae in nature, many of which possess medicinal, health-promoting, or cosmetic properties. Collectively, they are referred to as medicinal plants (Chao and Tong, 2022). A herbal garden is a specialized garden designed for the *ex situ* conservation, introduction, and domestication of medicinal plants, often simulating their native habitats and community structures (Zheng et al., 2018). These gardens serve multiple functions, including the preservation, research, and development of traditional medicinal plant germplasm, as well as education, science communication, cultural dissemination, recreation, and therapeutic applications (Lin et al., 2024).

Herbal gardens have a long and rich history. Their origins in China can be traced to ancient times, notably the legendary “Shennong Herbal Garden”, which is said to have existed approximately 2800 BC (Liao et al., 2023). Internationally, early examples include plant cultivation practices in ancient Egypt (2700 BC) and Babylon (2000 BC) (Qi, 2022). Today, these gardens have evolved into interdisciplinary hubs that integrate nature, culture, art, and science (Liao et al., 2023). For instance, in Hong Kong, 10 secondary schools have established herbal gardens dedicated to the conservation of Chinese medicinal herbs; these gardens now serve as vital educational resources for both secondary and tertiary institutions (Law et al., 2022). In Europe, botanical gardens function as public educational centers, playing a significant role in biomimetics education (Speck and Speck, 2023). In Northeast India, herbal gardens not only help conserve the biodiversity of local medicinal plant species but also provide professional training for residents in multiple fields, such as horticulture, agriculture, botany, forestry, landscaping, *ex situ* conservation, and environmental education (Mehra et al., 2023). In Argentina, migrants create medicinal plant gardens, transforming them into important sites for cultivation, care, health, and well-being practice (Kujawska and Jimenez-Escobar, 2023). Despite these diverse functions, research on the systematic planning and design of herbal gardens remains limited. Critical aspects, such as strategic species selection and the functional application of plant properties, lack comprehensive studies and evidence-based guidance, presenting key bottlenecks that require urgent attention.

This study takes the Herbal Garden of Guangzhou University of Chinese Medicine as a case study, analyzing the ecological habits, ornamental characteristics, medicinal functions, aromatic characteristics, and cultural significance of over 1,000 plant species. The objective is to offer practical guidance for institutions in the planning and design of such gardens, enabling them to maximize educational, research, and therapeutic potential while fostering healthy lifestyles and contributing to public health services.

## 2 Data

### 2.1 Study area

This study was conducted at the Herbal Garden of Guangzhou University of Chinese Medicine (GZUCM), hereafter referred to as the GZUCM Herbal Garden, located in Guangzhou, Guangdong Province (23°N, 113°E). Situated south of the Tropic of Cancer, Guangzhou experiences a southern subtropical monsoon climate characterized by warm temperatures, abundant rainfall, high solar radiation, long summers, and short frost periods. The city’s average annual temperature ranges from 21.7 °C to 23.1 °C, with an average annual precipitation of 1,923 mm and approximately 149 rainy days per year. The climatic conditions support lush, year-round vegetation, earning Guangzhou the nickname “City of Flowers”. Established in 2006, the GZUCM Herbal Garden covers a total area of 56,000 m^2^ and consists of the Shizhen Herbal Garden and the Yaowang Herbal Garden on the Panyu Campus, as well as the Sanyuanli Herbal Garden on the Baiyun Campus. It currently conserves more than 1,500 species of medicinal plants characteristic of the Lingnan region, including trees, shrubs, vines, and herbs. The GZUCM Herbal Garden serves as the “Germplasm Resource Nursery for the Fourth National Survey of Chinese Medicinal Resources in Guangdong Province” and the “Guangdong Key Species Conservation Nursery for Chinese Medicinal Plants”. It conserves a rich diversity of commonly used, regionally distinctive, and precious species identified during the resource survey and is considered representative across Guangdong Province in terms of both the quantity and variety of preserved species.

### 2.2 Data collection of medicinal plants

The GZUCM Herbal Garden has introduced and preserved approximately 1,500 species of medicinal plants. Due to limited documentation for some species, this study focused on 1,278 species with well-documented information available in the existing literature.

This study systematically surveyed, collected, and organized data on 1,278 medicinal plant species within the herbal garden. Authoritative sources, including *Flora of China* (Institute of Botany, Chinese Academy of Sciences, 2025), the *Chinese Pharmacopoeia* (Chinese Pharmacopoeia Commission, 2025), the *Guangdong Provincial Standards for Chinese Materia Medica* (Guangdong Provincial Food and Drug Administration, 2004), and *Chinese Materia Medica* (Hu et al., 1998), as well as online resources such as Baidu Encyclopedia, were comprehensively integrated to obtain foundational information for each species, which was then subjected to scientific analysis. The study covered taxonomic classification (family and genus), plant types, ecological habits, aromatic and ornamental characteristics, medicinal functions, edible properties, and cultural significance. Core information, particularly regarding medicinal functions, was strictly derived from regulatory standards such as the *Chinese Pharmacopoeia* and the *Guangdong Provincial Standards for Chinese Materia Medica*, ensuring the authority and reliability of the data. Subsequent chapters will build upon these survey results to conduct an in-depth and systematic analysis of medicinal plants in the herbal garden.

The raw and analyzed data have been shared in the form of attachments.

## 3 Plant characteristic analysis

### 3.1 Distribution of plant families and life forms

**Figure 1:**
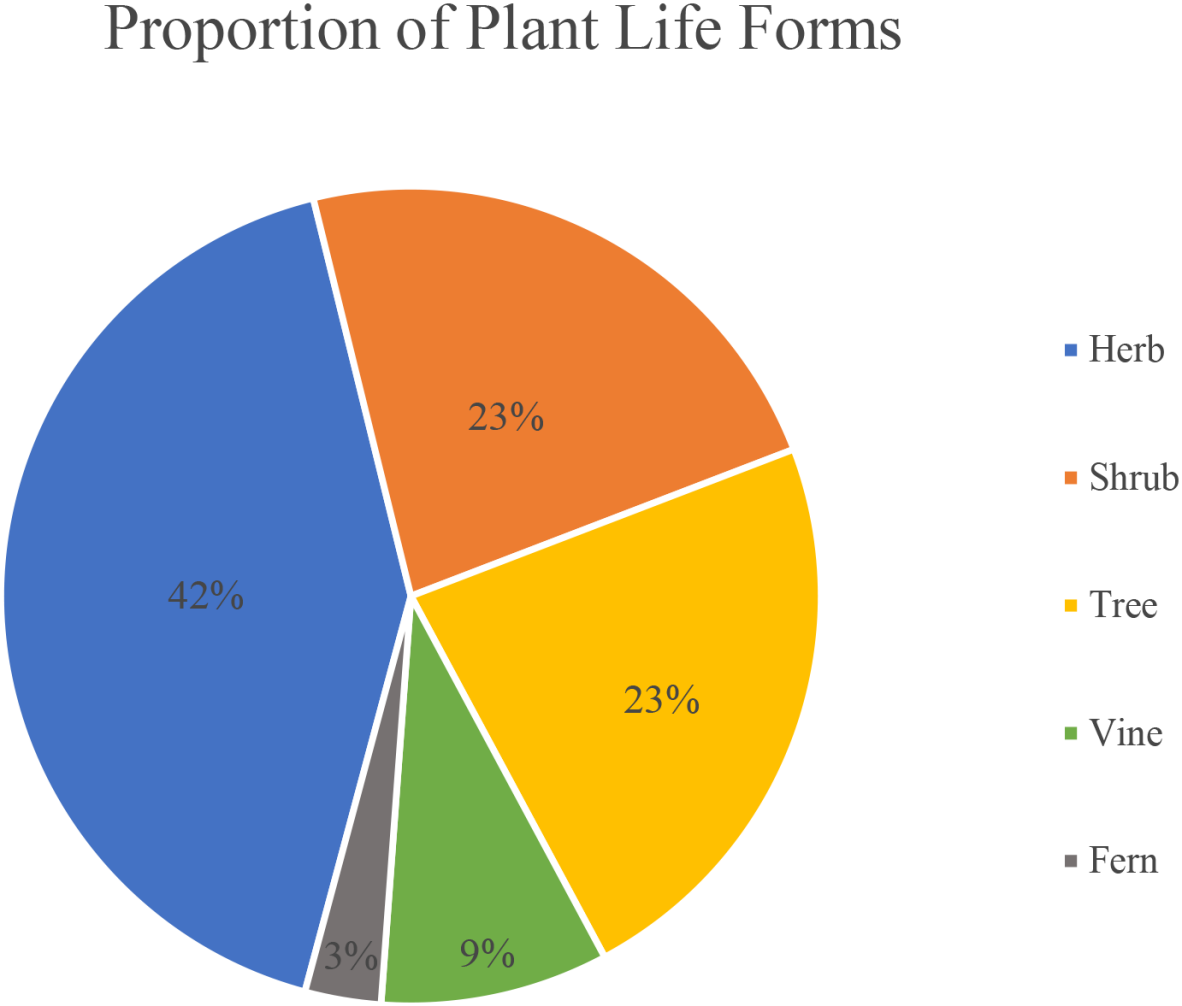
Proportion of various plant life forms (tabular data are available in Data Sheet 1).

**Figure 2:**
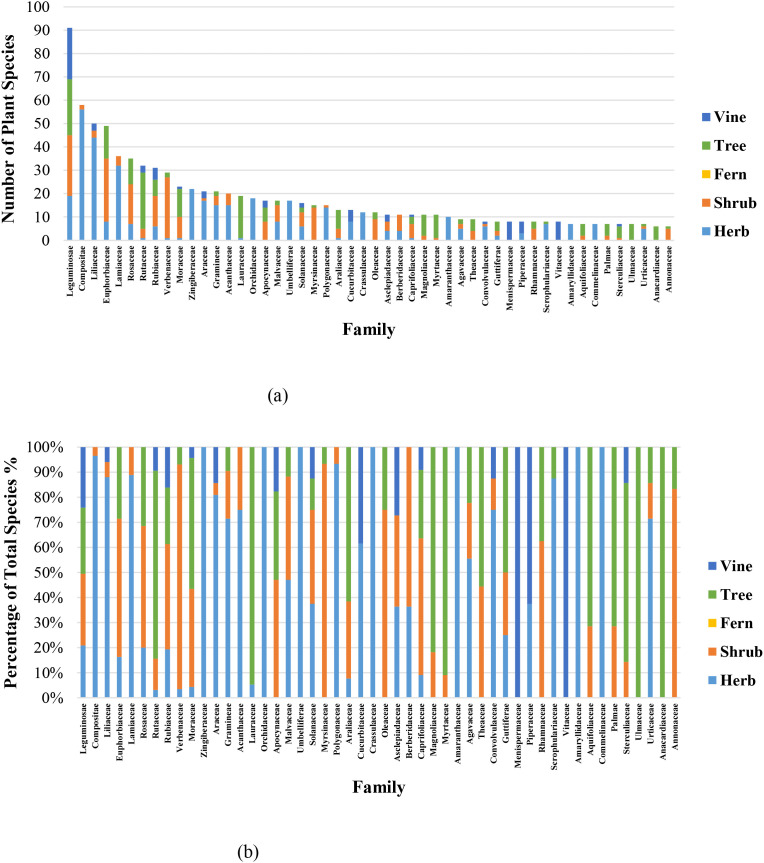
Life-form distribution among the 50 most species-rich families (tabular data are available in Data Sheet 2). **(a)** Number of species. **(b)** Percentage of total species.

A total of 1,278 medicinal plant species belonging to 190 families and 781 genera were recorded in the GZUCM Herbal Garden. Based on life-form classification, they include 533 herbs, 291 trees, 293 shrubs, 119 vines, and 42 ferns (**Figure 1**). The distribution of life forms among the 50 most species-rich families is shown in **Figure 2**.

Statistical data analysis of **Figures 1**, **2** highlights the garden’s high species diversity. In terms of life-form distribution, herbaceous plants are the most dominant, comprising 42% of the total, while trees and shrubs are nearly equal in abundance (each approximately 23%). Vines and ferns are relatively scarce, accounting for 9% and 3%, respectively. The proportion of herbaceous plants in our herbal garden (42%) is slightly lower than that reported (52.6%) for home gardens in the tropical rainforest climate of eastern Brazil (Pauletto et al., 2023). This discrepancy can likely be explained by the hotter and more humid conditions of the rainforest, which are more conducive to herbaceous plant growth compared to the subtropical monsoon climate of Guangzhou. Thus, our finding suggests a significant correlation between plant life-form distribution and regional climate. At the family level, the 10 most dominant families—Leguminosae, Compositae, Liliaceae, Euphorbiaceae, Lamiaceae, Rosaceae, Verbenaceae, Rutaceae, Rubiaceae, and Moraceae—collectively contain 447 species, representing 35% of all recorded species. These families form the core taxonomic composition of the garden’s vegetation.

### 3.2 Statistical analysis of ecological habits of medicinal plants

Ecological habits refer to the characteristic patterns, strategies, and behaviors of plants that have been shaped by natural selection in response to their environmental conditions (Liu et al., 2016). These traits reflect species-specific preferences for factors such as light, water, soil, and climate and form the basis for classifying plants into different ecological habit types.

Understanding the ecological habits of medicinal plants is critical for their successful introduction, cultivation, and conservation. In the application of medicinal plants, it is first necessary to understand their ecological types and habits. The selection of suitable species based on site-specific environmental conditions enhances survival rates and growth performance. Moreover, understanding the ecological preferences of medicinal plants helps us predict their growth performance under specific environmental conditions, enabling better planning and design of garden landscapes.

Temperature and soil pH are among the most influential environmental factors. Based on temperature preference, medicinal plants can be classified as cold-tolerant, semi-cold-tolerant, thermophilic, or heat-tolerant. According to soil pH requirements, they can be categorized as acidophilic, neutrophilic, or alkaliphilic (Zhu, 2019). Most medicinal plant gardens are geographically confined and share relatively homogeneous environmental conditions. The majority of species in the GZUCM Herbal Garden—located in a subtropical climate—are thermophilic and exhibit preferences for neutral to slightly acidic soils.

Light and water are critical ecological factors influencing plant growth and development. Based on light intensity preferences, plants can be categorized as heliophilic, shade-tolerant, or intermediate. According to water needs, they are classified as xerophytic, hygrophilous, mesophytic, or aquatic. In the GZUCM Herbal Garden, heliophilic species constitute 42.18%, intermediate species 40.38%, and shade-tolerant species 16.90%. A few species exhibit shade tolerance at the seedling stage and shift to light preference at maturity. Regarding water needs, mesophytic species account for 77.31% of the plants, hygrophilous species 17.29%, aquatic species 1.02%, and xerophytic species 4.38%. Overall, the majority of cultivated medicinal plants in the garden are heliophilic or intermediate in light requirements, and mesophytic or hygrophilous in water needs. As there is a wide variety of plant species in the garden, we have selected representative shade-tolerant, aquatic, and xerophytic medicinal plants for display, providing a reference for planting design (as detailed in **Tables 1**–**3**) (additional data are available via the link provided in Section 2.2).

**Table 1:** Common shade-tolerant medicinal plants.

No.	Plant species (Latin name)	Family	Life form	Medicinal part	Medicinal function
1	*Ophiopogon reversus* C. C. Huang	Liliaceae	Herb	Tuberous root	Nourishes yin, promotes fluid production, moistens the lungs, and clears the heart.
2	*Polygonatum cyrtonema* Hua	Liliaceae	Herb	Rhizome	Tonifies qi and yin, strengthens the spleen, moistens the lungs, and benefits the kidneys.
3	*Polygonatum odoratum* (Mill.) Druce	Liliaceae	Herb	Rhizome	Nourishes yin, moistens dryness, generates fluids, and quenches thirst.
4	*Reineckea carnea* (Andrews) Kunth	Liliaceae	Herb	Whole herb	Clears the lungs, relieves cough; cools the blood, stops bleeding; clears toxins, and relieves sore throat.
5	*Hosta plantaginea* (Lam.) Aschers.	Liliaceae	Herb	Flower, leaf, whole herb	Clears heat and removes toxins.
6	*Hosta albomarginata* (Hook.) Ohwi	Liliaceae	Herb	Flower, leaf, root	Cools the blood, stops bleeding, clears heat, and removes toxins.
7	*Paris polyphylla* var. *stenophylla* Franch.	Liliaceae	Herb	Rhizome	Clears heat, removes toxins, reduces swelling, and relieves pain.
8	*Ophiopogon bodinieri* H. Lév.	Liliaceae	Herb	Whole herb, tuberous root	Nourishes yin, moistens the lungs; benefits the stomach, promotes fluid production; clears the heart, and relieves irritability.
9	*Chlorophytum laxum* R. Br.	Liliaceae	Herb	Whole herb	Clears heat, removes toxins, reduces swelling, and relieves pain.
10	*Asparagus cochinchinensis* (Lour.) Merr.	Liliaceae	Herb	Tuberous root	Nourishes yin, moistens dryness, clears the lungs, and subdues internal heat.

**Table 2:** Common aquatic medicinal plants.

No.	Plant species (Latin name)	Family	Life form	Medicinal part	Medicinal function
1	*Oenanthe javanica* (Bl.) DC.	Umbelliferae	Herb	Whole herb, flower	Whole herb: clears heat, removes toxins; promotes urination; stops bleeding; lowers blood pressure.
2	*Alisma plantago-aquatica* L.	Alismataceae	Herb	Tuber	Promotes urination, drains dampness, clears heat, transforms turbidity, and lowers lipids.
3	*Typha angustifolia* L.	Typhaceae	Herb	Pollen	Stops bleeding, resolves blood stasis, and promotes urination to relieve stranguria.
4	*Typha orientalis* C. Presl	Typhaceae	Herb	Pollen	Stops bleeding, resolves blood stasis, and promotes urination to relieve stranguria.
5	*Saururus chinensis* (Lour.) Baill.	Saururaceae	Herb	Aerial part	Promotes diuresis, reduces swelling, clears heat, and removes toxins.
6	*Spirodela polyrhiza* (Linnaeus) Schleiden	Lemnoideae	Herb	Whole herb	Disperses wind-heat, promotes rash expression, and induces diuresis.
7	*Lemna minor* L.	Lemnoideae	Herb	Whole herb	Disperses wind-heat, promotes rash expression, and induces diuresis.
8	*Pistia stratiotes* L.	Araceae	Herb	Whole herb	Expels wind, promotes rash expression; induces diuresis, eliminates dampness; cools and invigorates blood.
9	*Acorus calamus* L.	Araceae	Herb	Rhizome	Warms the middle, reduces inflammation, and relieves pain.
10	*Sagittaria trifolia* subsp. *leucopetala* (Miq.) Q. F. Wang	Alismataceae	Herb	Corm, flower, aerial part	Invigorates blood, cools blood, clears heat, and removes toxins.
11	*Nelumbo nucifera* Gaertn.	Nymphaeaceae	Herb	Seed, plumule, radicle, receptacle, stamen, leaf, rhizome node	Seed: tonifies spleen, stops diarrhea, benefits kidney, astringes essence, nourishes heart, and calms the mind.
12	*Euryale ferox* Salisb. ex K. D. Koenig & Sims	Nymphaeaceae	Herb	Seed	Tonifies spleen and kidney, and astringes essence.
13	*Eleocharis dulcis* (Burm. f.) Trin. ex Hensch.	Cyperaceae	Herb	Corm	Clears heat, dissolves phlegm, and resolves food stagnation.

**Table 3:** Common xerophytic medicinal plants.

No.	Plant species (Latin name)	Family	Life form	Medicinal part	Medicinal function
1	*Schlumbergera truncata* (Haw.) Moran	Cactaceae	Shrub	Aerial part	Removes toxins and reduces swelling.
2	*Opuntia dillenii* (Ker Gawl.) Haw.	Cactaceae	Shrub	Root and stem, fruit, flower	Root and stem: promotes qi and blood circulation; cools blood to stop bleeding; removes toxins, and reduces swelling.
3	*Hylocereus undatus* (Haw.) Britt. et Rose	Cactaceae	Shrub	Stem, flower	Stem: relaxes tendons, activates meridians; removes toxins, and reduces swelling. Flower: clears heat, moistens lungs; relieves cough, and resolves phlegm.
4	*Epiphyllum oxypetalum* (DC.) Haw.	Cactaceae	Shrub	Flower, stem	Flower: clears lung heat, stops cough; cools blood, stops bleeding; nourishes heart, and calms the mind. Stem: clears heat and removes toxins.
5	*Opuntia ficus-indica* (L.) Mill.	Cactaceae	Tree	Root and stem	Clears lung heat, relieves cough; cools blood, and removes toxins.
6	*Hylotelephium erythrostictum* (Miq.) H. Ohba	Crassulaceae	Herb	Flower, whole herb	Flower: clears heat, eliminates dampness, improves vision, and relieves itching. Whole herb: clears heat, removes toxins, and stops bleeding.
7	*Echeveria secunda* Booth ex Lindl.	Crassulaceae	Herb	Whole herb	Clears heat, removes toxins, stops bleeding, and treats dysentery.
8	*Sedum bulbiferum* Makino	Crassulaceae	Herb	Whole herb	Clears heat, removes toxins, cools blood, stops bleeding, and treats malaria.
9	*Sedum emarginatum* Migo	Crassulaceae	Herb	Whole herb	Clears heat, removes toxins, cools blood, stops bleeding, and promotes urination.
10	*Sedum chauveaudii* Hamet	Crassulaceae	Herb	Whole herb	Clears heat, removes toxins, cools blood, stops bleeding, and promotes urination.

With regard to the plants in the herbal garden, shade-tolerant medicinal plants are predominantly herbs and ferns, while vines, shrubs, or trees are uncommon. The majority of these plants belong to families such as Liliaceae, Myrsinaceae, Orchidaceae, Zingiberaceae, and Araceae. Additionally, a few shade-tolerant species are found in Compositae, Schisandraceae, and Rutaceae. Aquatic medicinal plants are primarily herbaceous, with a concentration in families such as Alismataceae, Typhaceae, Araceae, Nymphaeaceae, Cyperaceae, and Lemnoideae. Xerophytic medicinal plants are mostly herbs, with a few shrubs and trees. These species are primarily found in families like Cactaceae, Crassulaceae, Agavaceae, Liliaceae, and Portulacaceae.

### 3.3 Statistical analysis of the functions of medicinal plants

Medicinal plants are utilized for their therapeutic and health-promoting properties. In China, these plants are processed through preparation and refinement to become Chinese medicinal herbs, which are then used under the guidance of traditional Chinese medicine (TCM) theory to prevent and treat diseases. TCM has a history spanning over 5,000 years and has developed into a distinctive system of medical theory, diagnosis, and therapeutic practices that are widely acknowledged worldwide. The efficacy of Chinese medicines refers to the generalization of their therapeutic actions and effects in disease prevention and treatment, as guided by TCM principles.

**Figure 3:**
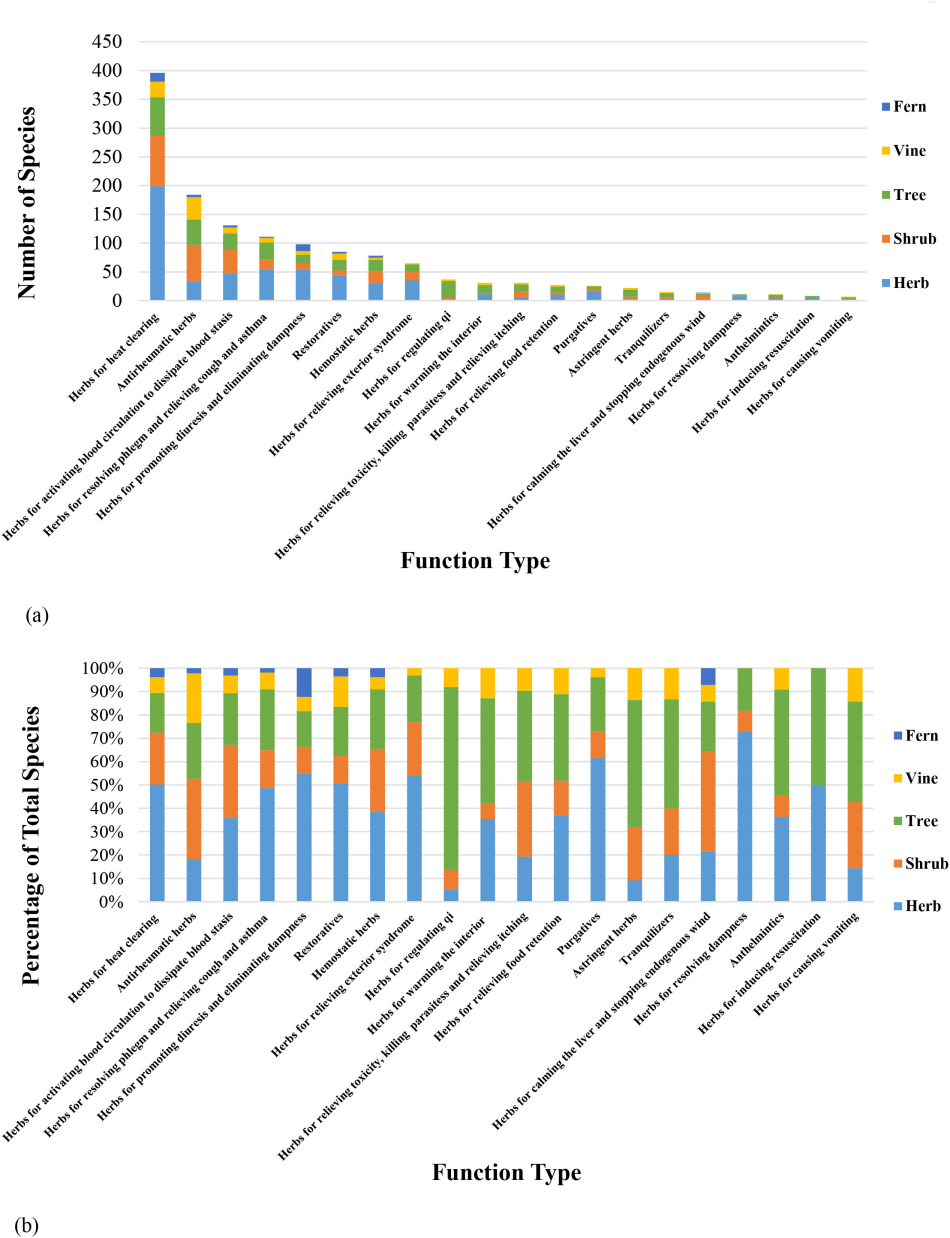
Distribution of medicinal plant species by function type. **(a)** Number of species of medicinal plants categorized by life form under each function type. **(b)** Proportion of species of medicinal plants categorized by life form under each function type.

Based on both the common and distinctive properties of Chinese medicinal herbs, including their nature, medicinal functions, indications, and contraindications, commonly used herbs can be classified into 20 major categories, including herbs for heat clearing, herbs for relieving exterior syndromes, purgatives, and antirheumatic herbs (Zhong and Yang, 2021). The distribution of plant species in the GZUCM Herbal Garden, categorized by their functions, is shown in **Figure 3**.

According to **Figure 3** (tabular data are available in Data Sheet 3), a total of 1,278 medicinal plants can be classified into 20 function types, encompassing 1,388 distinct plant species. It should be noted that some medicinal plants, when used from different plant parts, may have varying medicinal functions, resulting in the same plant being classified under multiple function types. For example, *Perilla frutescens* (L.) Britt. has the following medicinal parts and corresponding functions: the mature fruit is used for promoting qi, resolving phlegm, relieving cough and asthma, and promoting bowel movements; the leaves (or young shoots) are used for dispersing wind-cold, promoting qi circulation, and harmonizing the stomach; and the stem is used for regulating qi, harmonizing the middle energizer, relieving pain, and preventing miscarriage. Therefore, *P. frutescens* (L.) Britt. is categorized under “herbs for resolving phlegm and relieving cough and asthma”, “herbs for relieving exterior syndromes”, and “herbs for regulating qi”.

Among the 20 function types, “herbs for heat clearing” represent the largest group, with 396 species (28.53% of the total). This is followed by “antirheumatic herbs” and “herbs for activating blood circulation to dissipate blood stasis”, which account for 13.25% (184 species) and 9.44% (131 species), respectively. Most herbs for heat clearing are herbaceous, typically using aerial parts, branches, leaves, or whole herbs, whereas “antirheumatic herbs” and “herbs for activating blood circulation to dissipate blood stasis” are predominantly woody plants (shrubs or trees), commonly utilizing stems, branches, cores, barks, and roots. Among the medicinal plants in the garden, “herbs for heat clearing” constitute the largest proportion. This pattern is likely closely related to regional cultural factors, such as the widespread “herbal tea” tradition. Local communities tend to favor herbs with heat-clearing and detoxifying properties, which has prompted more extensive research, systematic introduction, and domestication of these plants. As a result, a relatively abundant diversity of heat-clearing Chinese medicinal herbs has been successfully cultivated.

### 3.4 Summaries of the edible properties of medicinal plants

Through long-term practical experience in daily life and production, it has been observed that some medicinal plants, with low toxicity, can be consumed regularly to enhance physical health and provide health benefits. These plants function as both medicines and foods, a concept known as “Medicine Food Homology (MFH)”. According to the Catalog of Substances Traditionally Used as Both Food and Medicinal Herbs, 106 species of medicinal plants have been identified as MFH (National Health Commission of China, State Administration for Market Regulation, 2024). The *Chinese Pharmacopoeia* notes that the primary plants for most medicinal herbs may involve multiple species. For example, Chen Pi (dried tangerine peel) is derived from *Citrus reticulata* Blanco and its cultivated varieties in the Rutaceae family (Chinese Pharmacopoeia Commission, 2025). Therefore, the number of medicinal plants suitable for use as MFH exceeds 106. Data analysis revealed that 164 medicinal plant species in the GZUCM Herbal Garden are categorized as MFH, as detailed in **Table 4**.

**Table 4:** Summaries of MFH.

No.	Plant species (Latin name)	Family	Life form	Main edible and medicinal parts	Function	Common methods of consumption
1	*Illicium verum* Hook. f.	Illiciaceae	Tree	Mature fruit	Warms the yang, disperses cold, regulates qi, and relieves pain.	Used as a flavoring spice
2	*Allium macrostemon* Bunge	Liliaceae	Herb	Bulb	Unblocks yang, disperses nodules, promotes qi, and relieves stagnation.	Boiled in porridge, stewed in soup
3	*Lilium brownii* var. *viridulum* Baker	Liliaceae	Herb	Fleshy scaly leaf	Nourishes yin, moistens the lungs, clears the mind, and calms the spirit.	Stir-fried, stewed, in porridge, desserts, tea, etc.
4	*Ophiopogon japonicus* (L. f.) Ker-Gawl.	Liliaceae	Herb	Tuberous root	Nourishes yin, promotes fluid production, moistens the lungs, and clears the heart to calm the mind.	Boiled, brewed as tea, in porridge, stewed in soup
5	*Polygonatum sibiricum* Delar. ex Redoute	Liliaceae	Herb	Rhizome	Tonifies qi, nourishes yin, strengthens the spleen, moistens the lungs, and benefits the kidneys.	Boiled in porridge, infused in alcohol
6	*Polygonatum odoratum* (Mill.) Druce	Liliaceae	Herb	Rhizome	Nourishes yin, moistens dryness, promotes fluid production, and quenches thirst.	Boiled, stewed in soup
7	*Aloe vera* (L.) Burm. f.	Liliaceae	Herb	Leaf or juice	Laxative, clears liver heat, kills parasites, and treats skin disorders.	Eaten raw or cooked, fresh juice applied to the skin for the anti-inflammatory effect, etc.
8	*Asparagus cochinchinensis* (Lour.) Merr.	Liliaceae	Herb	Tuberous root	Nourishes yin, moistens dryness, clears the lungs, and subdues internal heat.	Boiled in porridge, stewed in soup, brewed in wine
9	*Plantago asiatica* L.	Plantaginaceae	Herb	Whole herb	Clears heat, promotes urination to relieve stranguria, expels phlegm, cools blood, and removes toxins.	Brewed as tea, boiled in porridge
10	*Mentha canadensis* Linnaeus	Lamiaceae	Herb	Aerial part	Disperses wind-heat, clears head and eyes, induces perspiration, soothes throat, and relieves liver qi stagnation.	Brewed as tea, boiled in porridge

MFH, Medicine Food Homology.

### 3.5 Summaries of the aromatic characteristics of medicinal plants

Medicinal plants are rich in chemical constituents, and some exude distinctive aromatic scents. In herbal gardens, aromatic plants enhance the surrounding environment with their unique scents, enriching the sensory experience of visitors. Odors have significant effects on human psychology and emotions. Research has shown that pleasant and refreshing fragrances can stimulate various autonomic nerve cells, inducing excitement and significantly reducing negative emotions such as stress, fatigue, and depression (Quan and Shi, 2013; Liang, 2019; Wu, 2023). This study has identified 235 species of medicinal plants with notable odors, such as fragrant scents. Ten of the common species are detailed in **Table 5**.

**Table 5:** Summaries of medicinal plants with fragrance and other special odors.

No.	Plant species (Latin name)	Family	Life form	Odorous part	Function type
1	*Illicium verum* Hook. f.	Illiciaceae	Tree	Fruit	Herbs for warming the interior
2	*Lilium brownii* var. *viridulum* Baker	Liliaceae	Herb	Flower	Restoratives
3	*Lilium pumilum* DC.	Liliaceae	Herb	Flower	Restoratives
4	*Tulipa gesneriana* L.	Liliaceae	Herb	Flower	Herbs for promoting diuresis and eliminating dampness
5	*Hosta plantaginea* (Lam.) Aschers.	Liliaceae	Herb	Flower	Herbs for heat clearing
6	*Hosta albomarginata* (Hook.) Ohwi	Liliaceae	Herb	Flower, aromatic	Restoratives
7	*Lavandula angustifolia* Mill.	Lamiaceae	Shrub	Whole herb	Tranquilizers
8	*Leonurus japonicus* Houttuyn	Lamiaceae	Herb	Tuber, whole herb	Herbs for activating blood circulation to dissipate blood stasis
9	*Ocimum basilicum* L.	Lamiaceae	Herb	Tuber, whole herb	Herbs for relieving exterior syndromes
10	*Perilla frutescens* (L.) Britt.	Lamiaceae	Herb	Tuber, whole plant	Herbs for relieving exterior syndromes, herbs for regulating qi, and herbs for resolving phlegm and relieving cough and asthma

### 3.6 Summaries of the ornamental characteristics of medicinal plants

Vision is one of the most important human senses, with individuals being especially sensitive to color and spatial perception. Many medicinal plants possess notable ornamental qualities, which can be effectively utilized in the design of herbal gardens to enhance the experience of visitors. This study conducted a statistical analysis of plants with ornamental attributes in herbal gardens, considering various aspects such as flowers, leaves, fruits, forms, and branch structures. A total of 497 medicinal plant species exhibit significant ornamental value. We have highlighted the commonly found species to serve as a reference for the landscape planning and design of herbal gardens (detailed in **Table 6**).

**Table 6:** Summaries of the ornamental characteristics of medicinal plants.

No.	Ornamental attribute	Characteristics	Examples of medicinal plants
1	Ornamental flowers	Unique flower shape	*Orthosiphon aristatus* (Blume) Miq., *Clinacanthus nutans* (Burm. f.) Lindau, *Viburnum plicatum* Thunb. f. *tomentosum* (Miq.) Rehder, *Pseudognaphalium affine* (D. Don) Anderberg, *Eupatorium fortunei* Turcz., *Paris polyphylla* Sm., *Platycodon grandiflorus* (Jacq.) A. DC., *Leonurus japonicus* Houtt., *Taraxacum mongolicum* Hand.-Mazz.
		Brightly colored flowers	*Ixora chinensis* Lam., *Mussaenda pubescens* W. T. Aiton, *Tagetes erecta* L., *Helianthus annuus* L., *Sterculia lanceolata* Cav., *Camellia petelotii* (Merr.) Sealy, *Clerodendrum bungei* Steud., *Lantana camara* L., *Rosa chinensis* Jacq., *Yulania liliiflora* (Desr.) D. L. Fu, *Urena lobata* L., *Thevetia peruviana* (Pers.) K. Schum., *Brunfelsia brasiliensis* (Spreng.) L. B. Sm. & Downs
2	Ornamental leaves	Beautiful foliage shape	*Glechoma longituba* (Nakai) Kupr., *Morinda officinalis* F. C. How, *Persicaria hydropiper* (L.) Spach, *Ficus tinctoria* G. Forst. subsp. *gibbosa* (Blume) Corner, *Glochidion puberum* (L.) Hutch., *Liriodendron chinense* (Hemsl.) Sarg., *Cinnamomum cassia* (L.) D. Don, *Biancaea sappan* (L.) Tod., *Melicope pteleifolia* (Champ. ex Benth.) Hartley, *Heteropanax fragrans* (Roxb.) Seem., *Ranunculus sceleratus* L., *Mahonia bealei* (Fortune) Carr.
		Brightly colored leaves	*Dieffenbachia seguine* (Jacq.) Schott, *Antidesma bunius* (L.) Spreng, *Codiaeum variegatum* (L.) Blume, *Triadica cochinchinensis* Lour., *Excoecaria cochinchinensis* Lour., *Sansevieria trifasciata* Prain var. *laurentii* (De Wildem.) N. E. Brown, *Cordyline fruticosa* (L.) A. Chev., *Coleus scutellarioides* (L.) Benth., *Geranium wilfordii* Maxim., *Alpinia pumila* Hook.F., *Caladium bicolor* (Ait.) Vent.
3	Ornamental fruits	Unique fruit shape	*Camellia oleifera* Abel., *Duchesnea indica* (Andrews) Focke, *Ardisia crenata* Sims, *Strophanthus divaricatus* (Lour.) Hook. & Arn., *Artabotrys hexapetalus* (L. f.) Bhandari, *Artocarpus heterophyllus* Lam., *Citrus maxima* (Burm.) Merr., *Cassia fistula* L., *Canavalia gladiata* (Jacq.) DC., *Sarcandra glabra* (Thunb.) Nakai, *Markhamia stipulata* (Wall.) Seem. ex K. Schum. var. *kerrii* Sprague, *Palhinhaea cernua* (L.) Vasc. & Franco, *Citrus medica* L. ‘Fingered’
		Abundant fruit yield	*Clausena lansium* (Lour.) Skeels, *Kadsura longipedunculata* Finet & Gagnep., *Ilex rotunda* Thunb., *Callicarpa dichotoma* (Lour.) K. Koch, *Litchi chinensis* Sonn., *Dimocarpus longan* Lour., *Syzygium samarangense* (Blume) Merr. & L. M. Perry, *Syzygium cumini* (L.) Skeels, *Morus alba* L., *S. glabra* (Thunb.) Nakai
4	Ornamental form or branches	Large tree	*Taxus wallichiana* Zucc. var. *mairei* (Lemée & H. Lév.) L. K. Fu & Nan Li, *Tectona grandis* L. f., *Houpoea officinalis* (Rehder & E. H. Wilson) N. H. Xia & C. Y. Wu, *Cerbera manghas* L., *Alstonia scholaris* (L.) R. Br., *Archidendron clypearia* (Jack) I. C. Nielsen, *Ficus altissima* Blume, *Dracontomelon duperreanum* Pierre, *Platycladus orientalis* (L.) Franco, *Archontophoenix alexandrae* (F. Muell.) H. Wendl. & Drude
		Small tree	*Laurus nobilis* L., *C. lansium* (Lour.) Skeels, *Acronychia pedunculata* (L.) Miq., *Murraya exotica* L., *Bambusa vulgaris* Schrad. ex J. C. Wendland f. *vittata* (Riviere & C. Riviere) T. P. Yi, *Ligustrum lucidum* W. T. Aiton, *Ligustrum sinense* Lour., *Ardisia quinquegona* Blume, *Carmona microphylla* (Lam.) G. Don, *Caryota maxima* Blume ex Mart.
		Large shrub	*Ricinus communis* L., *Jatropha curcas* L., *Heptapleurum heptaphyllum* (L.) Y. F. Deng, *Justicia adhatoda* L., *Allamanda schottii* Pohl, *Hoya carnosa* (L. f.) R. Br., *Buxus bodinieri* H. Lév., *Viburnum sempervirens* K. Koch, *Abrus pulchellus* Wall. subsp. *cantoniensis* (Hance) Verdc., *Rhododendron mucronatum* (Blume) G. Don
		Small shrub or herb	*Eleutherococcus trifoliatus* (L.) S. Y. Hu, *Ardisia japonica* (Thunb.) Blume, *Ardisia mamillata* Hance, *Zornia gibbosa* Span., *Derris trifoliata* Lour., *Peucedanum praeruptorum* Dunn, *Centella asiatica* (L.) Urb., *Hydrocotyle sibthorpioides* Lam., *Cnidium monnieri* (L.) Spreng., *Canna indica* ‘Edulis’, *Rhapis excelsa* (Thunb.) A. Henry, *Bletilla striata* (Thunb. ex A. Murray) Rchb. f., *Begonia maculata* Raddi

### 3.7 Summaries of the cultural and educational attributes of medicinal plants

Medicinal plants are deeply intertwined with daily life and have accumulated rich cultural significance over centuries of use. In the process of displaying and introducing these plants, incorporating historical references, folk tales, poetry, and the philosophical principles of TCM (along with the latest advancements in medicinal plant research, innovations, and health and wellness concepts) enhances the cultural attributes of medicinal plants. Integrating these cultural and educational aspects into the design and planning of herbal gardens can significantly enrich their educational value, reflect the distinctive features and values of traditional Chinese culture, deepen the cultural connotations of the herbal garden, and enable visitors to engage with both traditional TCM culture and the modern scientific developments in medicinal plants. This integration enhances the appeal and educational potential of the garden, making it a more attractive destination. Statistics on the cultural and educational attributes of medicinal plants are presented in **Table 7**.

**Table 7:** Summaries of cultural and educational attributes of medicinal plants.

No.	Attribute classification	Examples of medicinal plants	Effect/significance
1	Historical references and legends	*Leonurus japonicus* Houtt., *Plantago asiatica* L., *Amomum villosum* Lour., *Saposhnikovia divaricata* (Turcz.) Schischk., *Senna tora* (L.) Roxb., *Pleuropterus multiflorus* (Thunb.) Nakai, *Angelica dahurica* (Fisch. ex Hoffm.) Benth. & Hook. f. ex Franch. & Sav., *Citrus reticulata* ‘Chachiensis’, *Lycopodium japonicum* Thunb., *Clematis chinensis* Osbeck, *Vincetoxicum pycnostelma* Kitag., *Reynoutria japonica* Houtt., *Salvia miltiorrhiza* Bunge, *Schisandra chinensis* (Turcz.) Baill.	These plants are introduced through accessible stories, helping the public better understand and familiarize themselves with medicinal plants.
2	Poetry, songs, and literary references	*Nelumbo nucifera* Gaertn., *Chrysanthemum* × *morifolium* (Ramat.) Hemsl., *Prunus mume* Siebold & Zucc., *Cymbidium* Sw., Bambusoideae, *Kaempferia galanga* L., *Saururus chinensis* (Lour.) Baill.	Demonstrates the integration of TCM with Chinese culture, showcasing the depth and breadth of traditional Chinese culture.
3	Research and innovation achievements	*Artemisia annua* L., *Pogostemon cablin* (Blanco) Benth., *Aquilaria sinensis* (Lour.) Spreng., *A. villosum* Lour.	Highlights the advanced results of modern research in TCM, showcasing innovative developments.
4	Health and wellness concepts	Medicinal food formulas: WALOVI herbal tea, Xiasangju herbal tea	Promotes the concept of “preventive treatment of diseases” in TCM, integrating health and wellness ideas.
5	Interactivity	*Mimosa pudica* L., *Plectranthus*‘Cerveza’n Lime’, *Taraxacum mongolicum* Hand.-Mazz., *Mentha canadensis* L., *Ocimum basilicum* L., *Mentha* sp*icata* L., *Foeniculum vulgare* Mill., *Rosmarinus officinalis* L., *P. cablin* (Blanco) Benth., *Agastache rugosa* (Fisch. & C. A. Mey.) Kuntze, *Houttuynia cordata* Thunb., *Piper sarmentosum* Roxb., *Ruta graveolens* L., *Cymbopogon citratus* (DC.) Stapf, *Perilla frutescens* (L.) Britt.	Encourages sensory interaction with medicinal plants through touch, smell, and other sensory experiences, enhancing visitor engagement and the effectiveness of scientific education.

TCM, traditional Chinese medicine.

It is evident that, beyond their medicinal and ornamental functions, medicinal plants possess significant cultural and educational attributes. These attributes can be effectively integrated into the planning and design of herbal gardens, thereby enhancing the depth and value of the garden’s educational and cultural offerings.

## 4 Apply policy

Medicinal plants are diverse, and in the planning and design of herbal gardens, it is essential to consider the local context, including climate, soil conditions, sunlight, local culture, and other factors. The characteristics of plants should be analyzed to create gardens that are not only attractive and sustainable but also tailored to meet the specific needs of the region. The author’s team has contributed to the design and planning of herbal garden projects at institutions such as Guangzhou University of Chinese Medicine (in Guangzhou City), Guangdong Agribusiness Tropical Agriculture Institute Co., Ltd. (in Guangzhou City), Fogang Town Central Primary School (in Qingyuan City), Fogang Town Sanatorium (in Qingyuan City), Kunpeng Middle School (in Yunfu City), TCM Hall (in Dongguan City), Tsinglan Primary School (in Dongguan City), and Guangdong Provincial Southern Medicinal Seed Industry Innovation Park (in Maoming City). Based on practical experience, we offer strategic recommendations for the planning and design of herbal gardens.

### 4.1 Adhere to ecological principles to preserve biodiversity

In the broader sense, biodiversity encompasses three levels: ecosystem diversity, species diversity, and genetic diversity (The State Council Information Office of the People’s Republic of China, 2021). As a localized ecological area, the focus of biodiversity in herbal gardens primarily concerns species diversity and habitat diversity.

#### 4.1.1 Maintain the diversity of medicinal plant species

When introducing new species, efforts should be made to enrich the range of plant families and life forms, thereby maximizing plant population diversity. Plants have distinct ecological habits, and in the planning and design of the herbal garden, it is essential to understand the ecological characteristics of different plant populations. This enables the selection of species that minimize interspecific competition, prevent the formation of monocultures, foster mutually beneficial symbiotic relationships, and contribute to a stable micro-ecosystem in the garden. In the design process, it is crucial to plan for a variety of life forms, including tall trees, small trees, shrubs, herbs, and vines. This ensures a balanced distribution of plants both vertically and horizontally across the space. The arrangement of a diverse range of species that bloom and fruit in different seasons will create a dynamic seasonal landscape. A scientifically designed layout that incorporates plants with complementary needs (e.g., heliophilic, shade-tolerant, intermediate, xerophytic, hygrophilous, mesophytic, and aquatic plants) optimizes resource allocation and encourages mutual coexistence among species. Therefore, a scientifically informed approach to plant introduction and species diversity is essential for ensuring the quality of medicinal plant resources, maintaining ecosystem balance, preventing monotony, and enhancing the visitor experience with the diverse wonders of the plant world.

#### 4.1.2 Maintain habitat diversity

The climate varies seasonally depending on the latitude and longitude of the herbal garden’s location. Prior to planning the garden, a thorough site survey and investigation are essential to understand the local climate, including temperature, sunlight, precipitation (moisture), soil pH, fertility, and composition. This knowledge is crucial to understanding the overall ecological conditions of the herbal garden. Only by selecting plants that are well-suited to the local environment can we successfully introduce, conserve, and propagate species, ensuring the sustainability of plant resources. Additionally, diverse habitats should be created within the garden. First, observe the light intensity and duration, and any potential shading from buildings or tall trees. Based on this, select plants that thrive in different light conditions—heliophilic, shade-tolerant, or intermediate. For example, shade-tolerant plants can be planted under dense tree canopies. Second, observe the terrain to select suitable plants: flat areas are ideal for herbs or shrubs; low-lying or waterlogged zones can be designated for aquatic plants.

**Figure 4:**
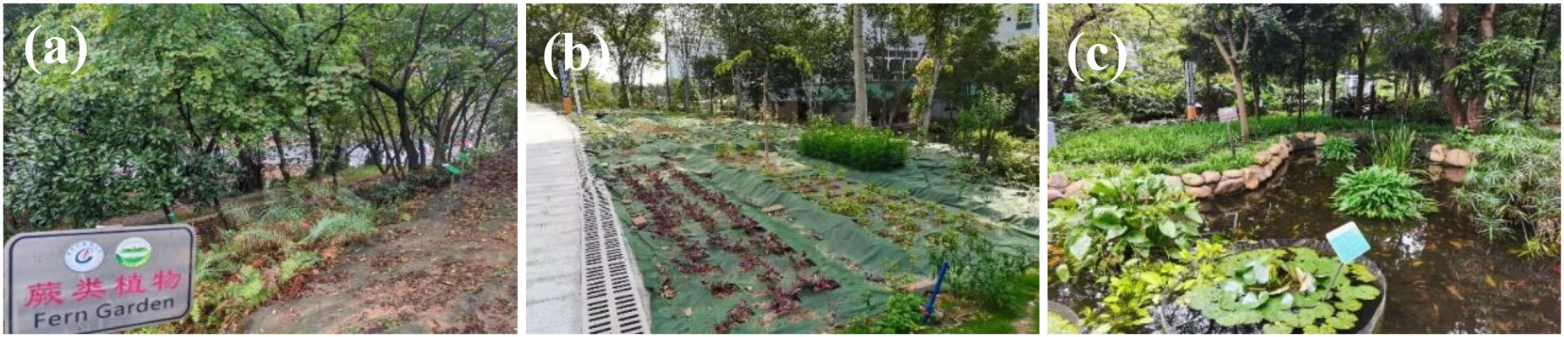
Local area in the GZUCM Herbal Garden. **(a)** Fern planting area. **(b)** Herb planting area. **(c)** Aquatic planting area. GZUCM, Guangzhou University of Chinese Medicine.

In a limited space, creating varied habitats allows for the display of a broader range of plant species, enhancing biodiversity and creating a visually dynamic landscape. For example, at the GZUCM Herbal Garden, shade-tolerant ferns were planted under a dense tree forest on the eastern slope; heliophilic herbs were placed on the sunny, gentle slopes; and the aquatic plant area combined ponds with plantings of tall trees, shade-tolerant species, and aquatic plants to create rich habitats and scenic views (**Figure 4**).

### 4.2 Adhere to health and safety principles

**Figure 5:**
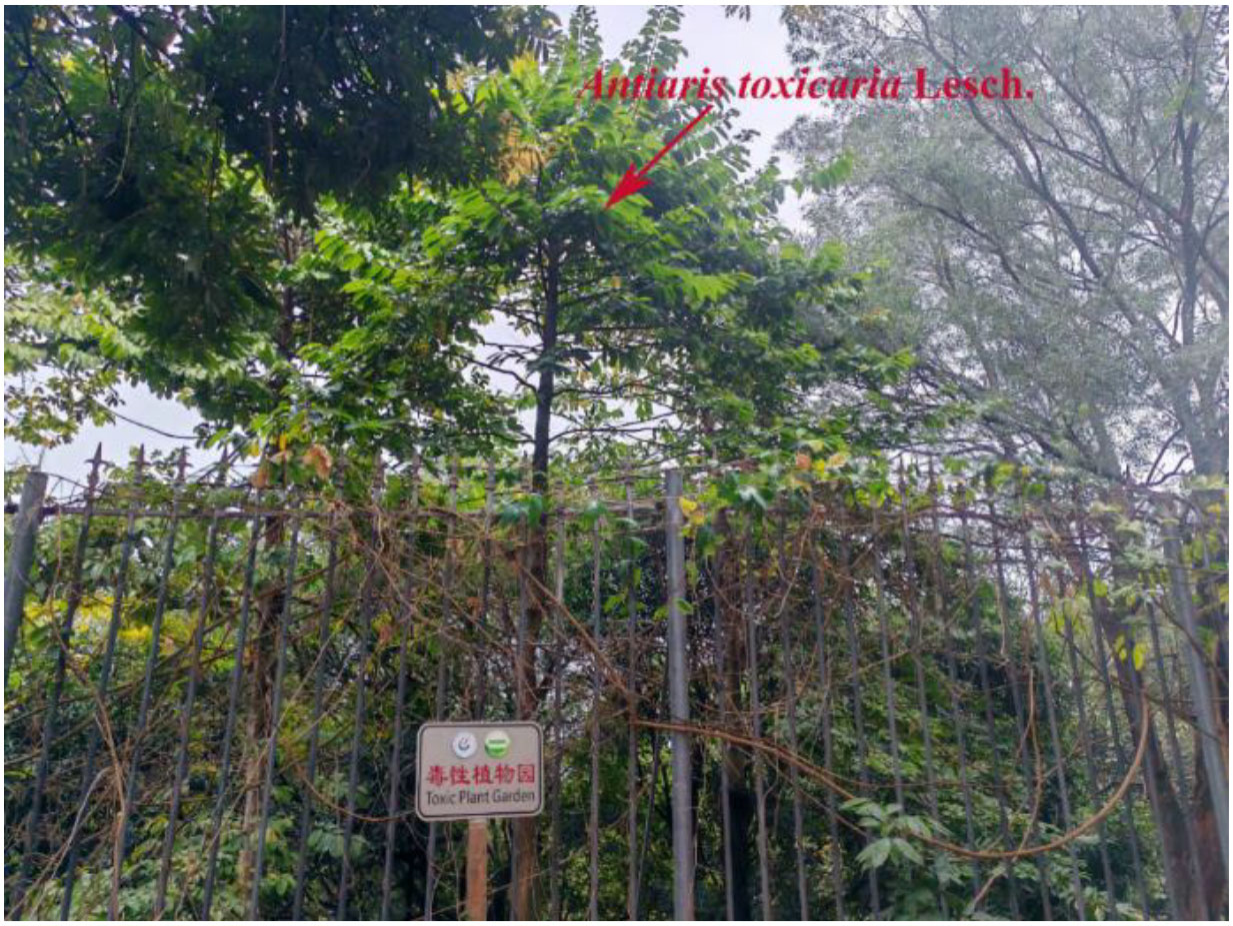
Toxic plant garden (Shizhen Herbal Garden in GZUCM). GZUCM, Guangzhou University of Chinese Medicine.

**Figure 6:**
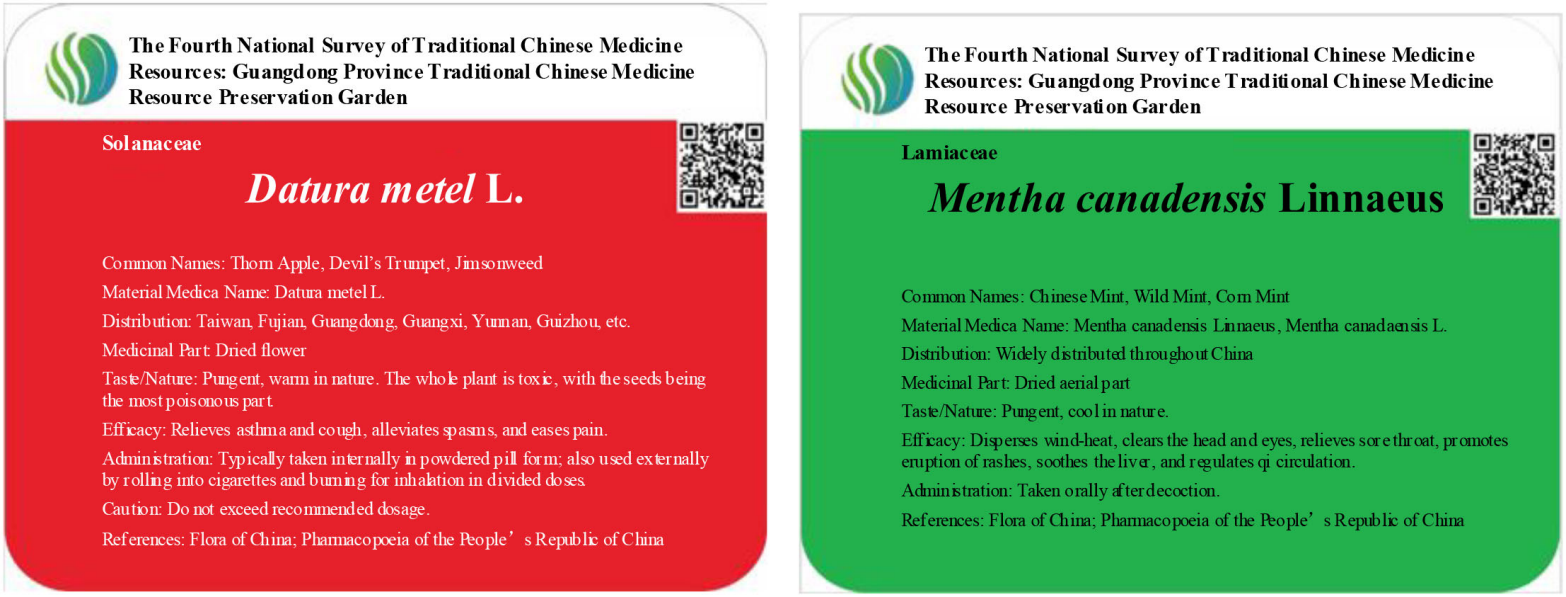
Medicinal plant signage.

Certain medicinal plants contain toxic substances that may cause harm or even death upon contact. These plants are referred to as “toxic plants”. In the design of a herbal garden, careful planning is necessary to designate specific areas for toxic plants, along with implementing protective measures and appropriate warning signage. Plants with minor toxicity but ornamental value can be planted away from pedestrian pathways, allowing visitors to admire them without easy access. Examples include species like *Euphorbia milii* Des Moul. and *Catharanthus roseus* (L.) G. Don. For more toxic plants, stringent safety measures are required to prevent accidental handling or ingestion. For instance, *Antiaris toxicaria* Lesch., known for its deadly latex, produces a sap so toxic that even minimal contact with the bloodstream can be fatal to both humans and animals. It should be planted with appropriate barriers, such as guardrails, to prevent contact (**Figure 5**). It is also essential to clearly distinguish between toxic and non-toxic plants in the garden, ensuring proper signage and warnings to alert visitors to potential hazards. In the GZUCM Herbal Garden, plant signage follows a color-coding system: green signs are used for general non-toxic plants, while red signs indicate toxic plants, ensuring that visitors are aware of safety precautions (**Figure 6**).

### 4.3 Scientific zoning design based on the purpose and scale of the herbal garden, following the principle of practicality

The key factors in the planning of a herbal garden include the construction objectives, park scale, financial guarantee, site selection, and environmental conditions. A scientific layout should be based on these objective factors to ensure optimal functionality. For example, the garden’s layout can be designed with scientifically zoned plant areas to effectively serve multiple functions, such as education, popular science dissemination, and rehabilitation, thereby maximizing its overall benefits. Some zoning design proposals are provided below for reference.

#### 4.3.1 Zoning based on plant taxonomy principles

**Figure 7:**
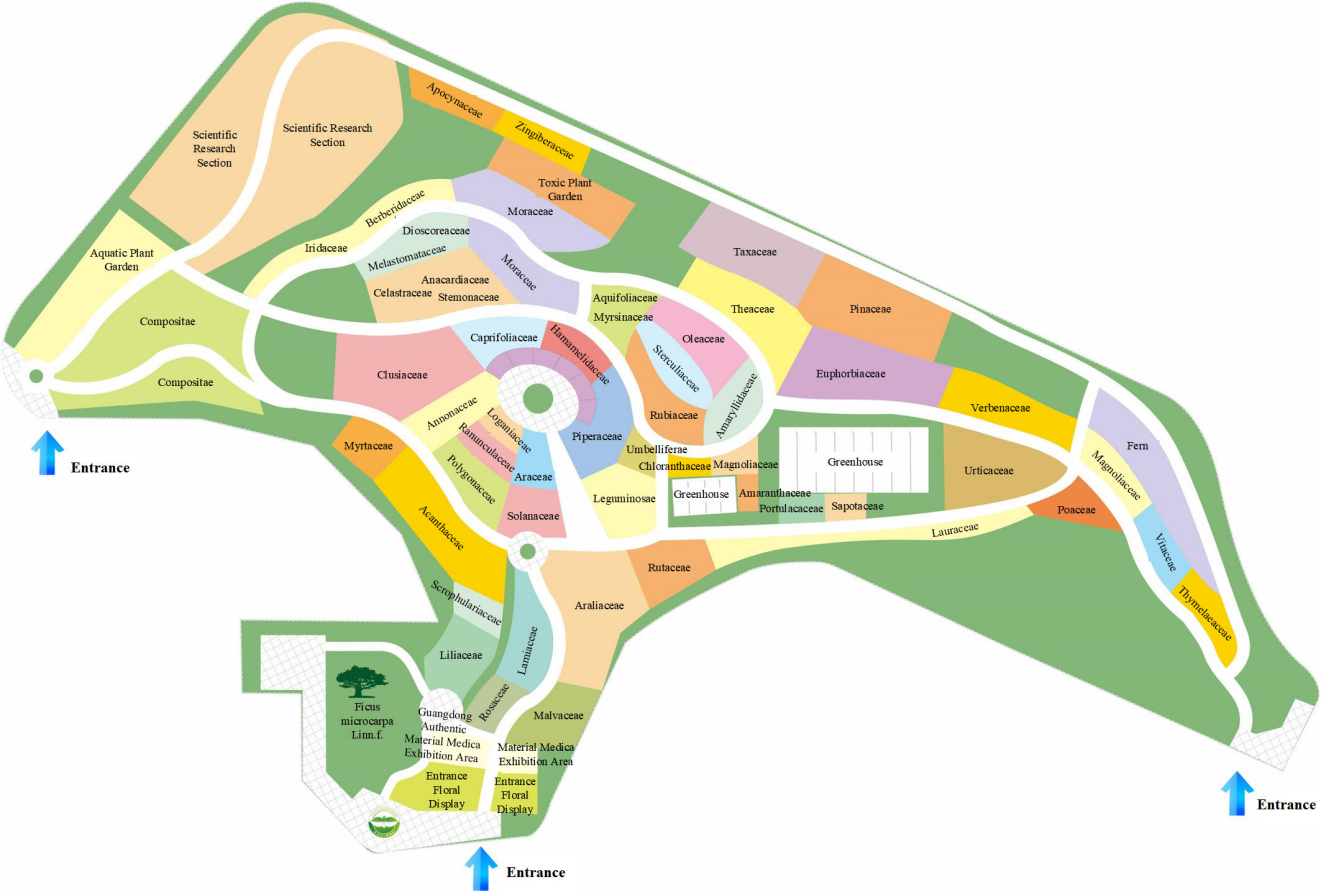
Zoning map of Shizhen Herbal Garden in GZUCM. GZUCM, Guangzhou University of Chinese Medicine.

There is a vast diversity of plant species on Earth, with estimates exceeding 500,000. Plant taxonomy enables the grouping, classification, and systematic arrangement of the highly complex plant kingdom, facilitating the understanding and utilization of plants. Educational institutions aiming to showcase the characteristics of plants from different families and highlight their similarities and differences may consider zoning based on plant taxonomy principles. For example, the Shizhen Herbal Garden in GZUCM follows Engler’s plant classification system (Engler and Prantl, 1897) and is divided into plant families, including Lamiaceae, Verbenaceae, Leguminosae, and Liliaceae (**Figure 7**). This zoning provides an excellent practical resource for courses such as *Medicinal Botany* and *Identification of Traditional Chinese Medicine*.

#### 4.3.2 Zoning based on the medicinal function of medicinal plants

**Figure 8:**
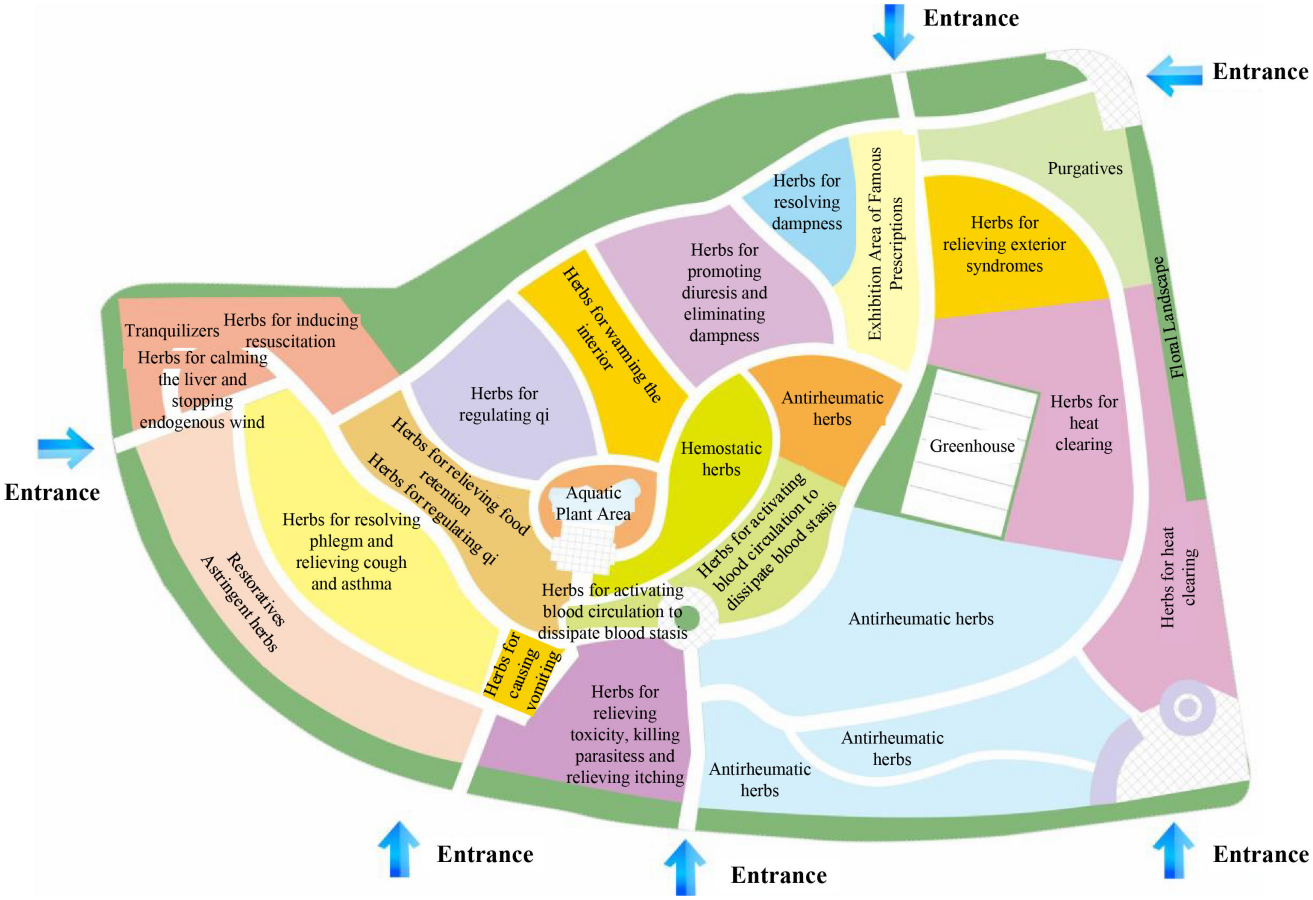
Zoning map of Yaowang Herbal Garden in GZUCM. GZUCM, Guangzhou University of Chinese Medicine.

Medicinal plants are those with therapeutic properties. As discussed in Section 3.3, medicinal plants can be categorized into 20 major groups based on their medicinal properties and functions. Guangdong is one of China’s four major medicinal material production regions, renowned for its variety of “Southern Medicine” and famous “Guangdong Medicine” (Xu et al., 2004). At the Yaowang Herbal Garden in GZUCM, guided by TCM principles, the garden is divided into 20 zones based on the medicinal function of the plants (**Figure 8**), showcasing the diverse functions of “Southern Medicine” and “Guangdong Medicine”. Classifying medicinal plants by their functions helps educate the public on the characteristics and medicinal functions of these plants, guiding their rational use while promoting health and wellness principles to enhance the effectiveness of public health education and scientific dissemination.

#### 4.3.3 Zoning based on the edible properties, aromatic characteristics, and interactive features of medicinal plants

**Figure 9:**
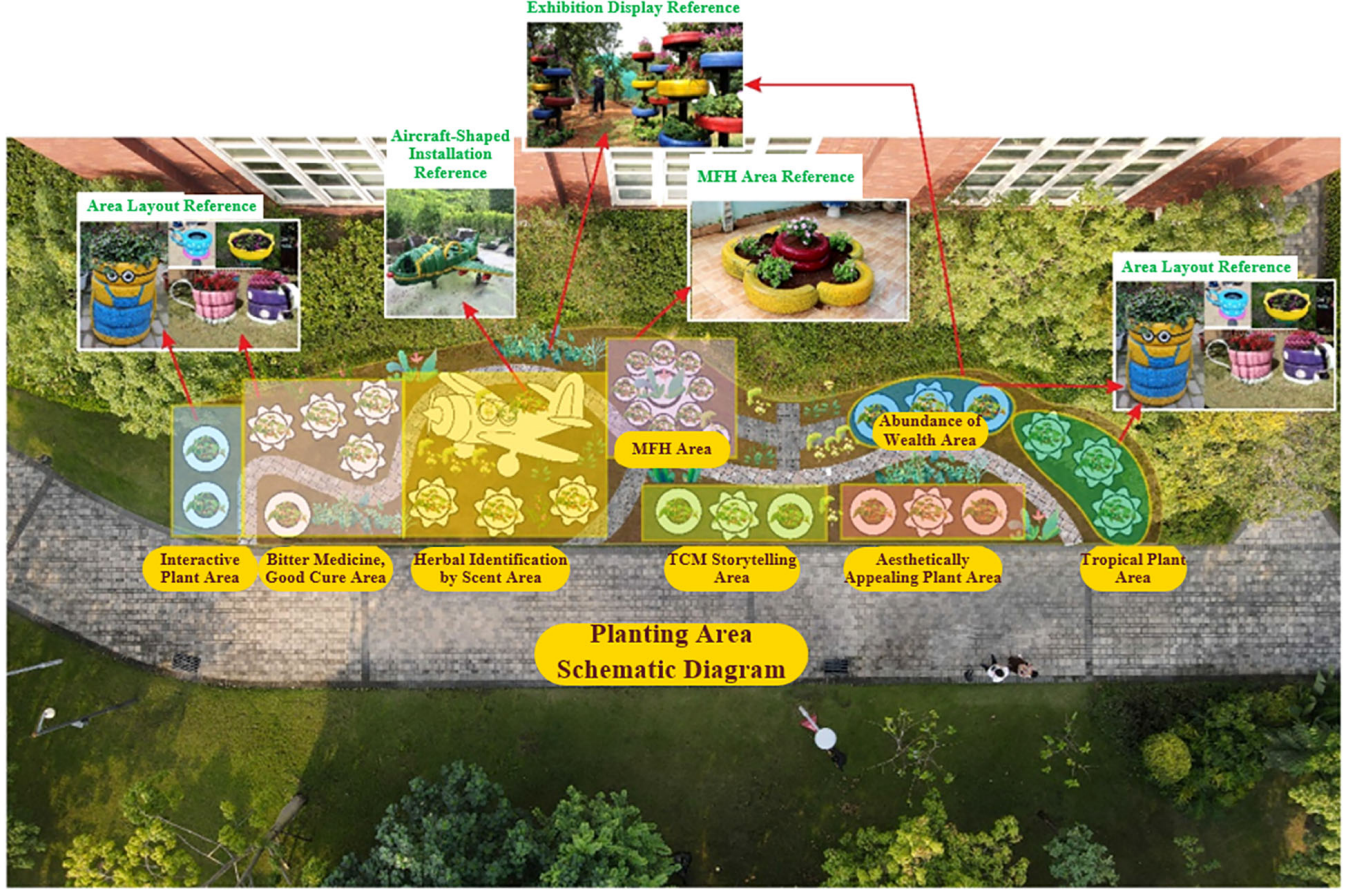
Medicinal plant garden design at Tsinglan Primary School.

In smaller-scale medicinal plant gardens, such as those in communities, parks, and educational institutions, where the number of plant species is limited, it is beneficial to focus on plants that can spark public interest. Sections 3.4, 3.5, and 3.7 analyzed the edible properties, aromatic characteristics, and cultural/educational features of medicinal plants, respectively. It is possible to create dedicated areas that highlight plants with culinary uses, aromatic characteristics, or interactive features. For example, when designing the herbal garden at Qinglanshan Primary School in Dongguan, functional areas were created to match the lively, curious, and energetic nature of primary school students. These included the “Interactive Plant Area”, the “Bitter Medicine, Good Cure Area”, “Herbal Identification by Scent Area”, and the “MFH Area”. The “Interactive Plant Area” features medicinal plants such as *Mimosa pudica* L., *Plectranthus* ‘Cerveza’n Lime’, and *Taraxacum mongolicum* Hand.-Mazz., which respond to touch. When students gently touch the leaves of *M. pudica* L., they rapidly close. Teachers can use this phenomenon to explain the plant’s sensitivity mechanisms and its intriguing historical use for predicting weather. The “Bitter Medicine, Good Cure Area” includes plants such as *Andrographis paniculata* (Burm. F.) Nees, which are bitter yet non-toxic. Students can taste the leaves to directly experience the principle of “Bitter Medicine, Good Cure”, reinforcing the traditional medicinal concept. The “Herbal Identification by Scent Area” is planted with aromatic medicinal herbs such as *Mentha canadensis* Linnaeus, *Ocimum basilicum* L., and *Mentha* sp*icata* L., guiding students to recognize herbs by their unique scents and enhancing sensory memory and botanical knowledge. The “MFH Area” features common herbs like *Lilium brownii* var. viridulum Baker and *Ophiopogon japonicus* (L. f.) Ker-Gawl., which are used as both food and medicine. This area introduces the concept of “food therapy”, helping students develop an early understanding of healthy living (**Figure 9**).

When planning a herbal garden, the planting layout can be enriched by incorporating cultural elements such as historical references, folk legends, and classical Chinese poetry. Through educational interpretation, these elements help highlight the cultural attributes of medicinal plants and create a culturally immersive atmosphere. For instance, the etymology of plant names and the discovery of their medicinal functions often have fascinating historical and folkloric backgrounds. Examples such as the origin of the name “Motherwort (*Leonurus japonicus* Houttuyn)” or the discovery of the medicinal functions of *Amomum* (*Amomum villosum* Lour.) are rooted in historical narratives that can spark public curiosity. Additionally, plants like the *Nelumbo nucifera* Gaertn., *Chrysanthemum × morifolium* (Ramat.) Hemsl., *Prunus mume* Siebold & Zucc., and *Cymbidium* Sw., which frequently appear in ancient Chinese poetry, are often used to symbolize noble qualities, reflecting the ancient literati’s admiration for virtue. By integrating such cultural references into scientific education, the public can be encouraged to adopt values of integrity and virtue, promoting a healthy societal outlook. Moreover, the garden can also serve as a platform to showcase the latest advancements in medicinal plant research, demonstrating their scientific applications and encouraging primary and secondary school students to study hard and pursue innovation.

#### 4.3.4 Incorporating regional characteristics to establish designated zones for geo-authentic medicinal materials, bulk herbs, and plants commonly used in traditional formulas

Geo-authentic medicinal materials refer to medicinal plants of superior quality and efficacy, produced in specific regions, known for their higher quality and more stable efficacy compared to those of the same species from other areas. Bulk herbs are widely demanded varieties in the TCM industry. Given the distinct regional distribution patterns of medicinal plants, targeted efforts can be made to introduce and cultivate geo-authentic medicinal materials and the source plants of commonly used bulk herbs in the herbal garden, thereby promoting the scientific conservation of plant resources and showcasing their industrial applications.

For example, in Huazhou, a renowned source of geo-authentic medicinal materials, a “Huazhou *Citrus maxima* ‘Tomentosa’ Germplasm Resource Nursery” was established to collect different germplasms of Huazhou *C. maxima* ‘Tomentosa’ plants for the preservation and exhibition of plant germplasms. This not only ensures the preservation of key local germplasm but also offers a platform to showcase the development of TCM, the medicinal plant industry, and its distinctive features.

**Figure 10:**
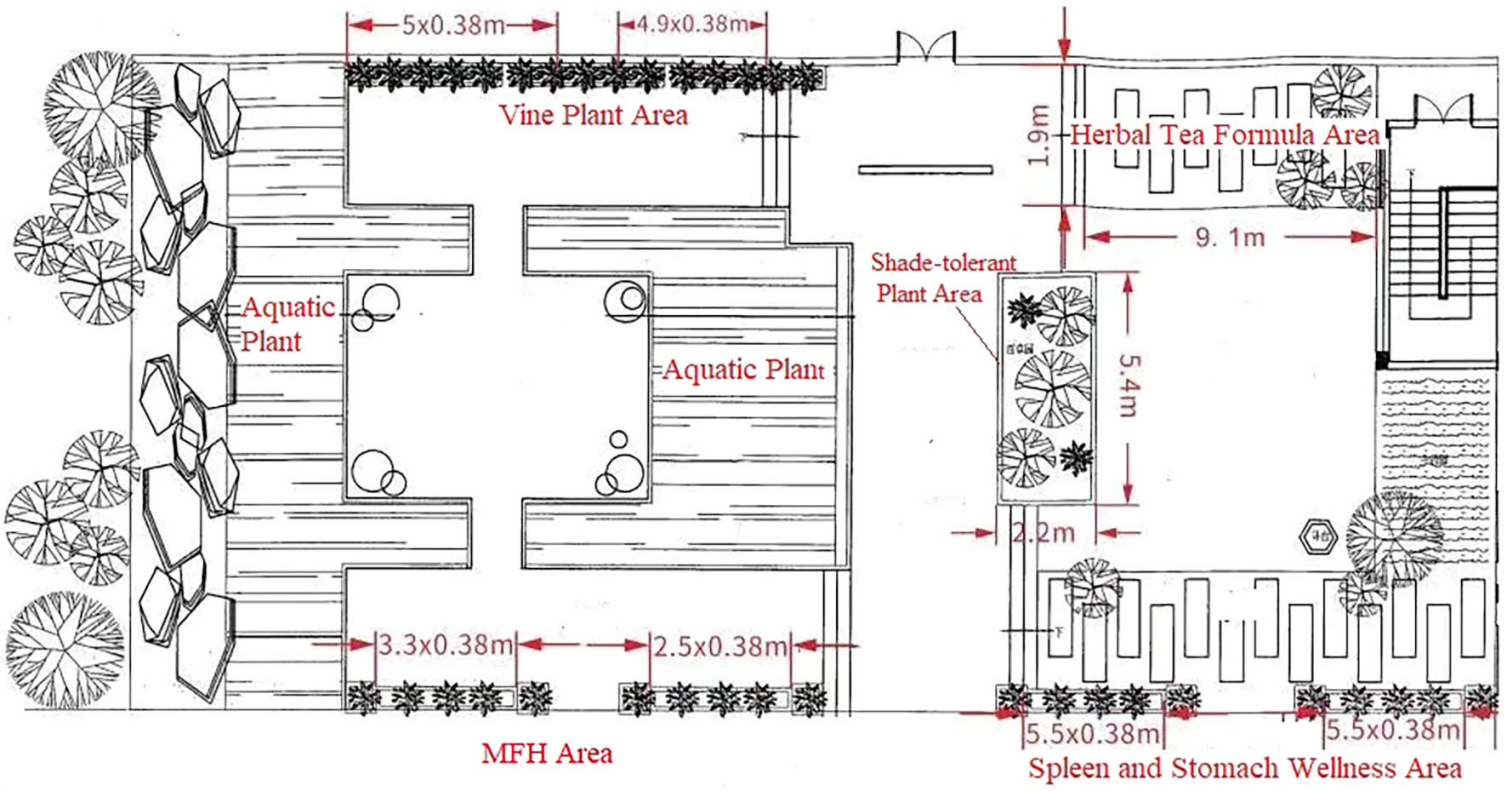
Design of the TCM Hall Herbal Garden. TCM, traditional Chinese medicine.

Additionally, the herbal garden can highlight plants related to local dietary habits by displaying the original plants of widely consumed herbal formulas. In Guangdong, where herbal teas such as Denglao Herbal Tea, WALOVI, and Xiasangju are commonly consumed, the garden can showcase the source plants of these popular formulas. This exhibition educates the public about the plant origins of these herbal teas and promotes the TCM concept of “preventive treatment of diseases”, guiding people toward a health-conscious lifestyle. When designing the herbal garden for TCM Hall, we took into account that the majority of visitors are patients seeking treatment. According to incomplete statistics, over 80% of these visitors suffer from gastrointestinal or upper respiratory conditions. In response, we specifically created the “Spleen and Stomach Wellness Area” and the “Herbal Tea Formula Area”, concentrating on the display of relevant Chinese medicinal herbs. These areas aim to deepen the public’s understanding of TCM knowledge in preventing and treating gastrointestinal and respiratory ailments, promote the concept of “preventive treatment of diseases”, and guide visitors toward strengthening daily health maintenance. Subsequent follow-up visits have shown that the garden is widely welcomed by both patients and nearby residents, with daily visitor numbers more than doubling compared to previous levels. This has effectively enhanced public awareness, understanding, and interest in TCM (**Figure 10**).

### 4.4 Selecting a planting scheme that adapts to the site conditions

**Figure 11:**
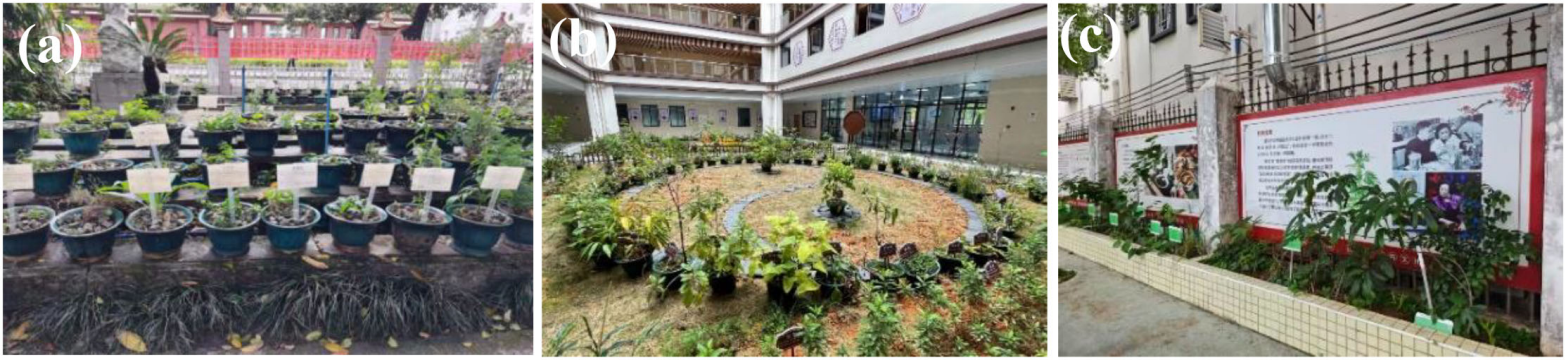
Photographs of selected herbal gardens. **(a)** Sanyuanli Herbal Garden in GZUCM. **(b)** Sanatorium Herbal Garden. **(c)** Fogang Primary School Herbal Garden. GZUCM, Guangzhou University of Chinese Medicine.

When constructing herbal gardens, site-specific limitations may prevent the planting of all desired species directly in the ground. For example, in smaller spaces such as residential communities, primary and secondary schools, or hospitals, it may be necessary to adapt planting methods based on available space. In such cases, flexible solutions such as the use of flower pots, raised beds, and ground planting can be employed. When selecting flower pots or raised beds, it is essential to ensure adequate water and air permeability, while also considering proper plant density to support healthy growth. For ground planting, ridge planting can be utilized (forming raised platforms at specific widths and intervals within the planting area) by piling soil into “ridges”, which enhances soil permeability, ensures proper drainage, and prevents soil compaction that may hinder plant growth. At our Sanyuanli Herbal Garden and the Qingyuan Fogang Sanatorium Herbal Garden, both of which have limited space, we primarily use potted plants to increase the variety of species on display (**Figures 11a, b**). At Fogang Primary School in Qingyuan, medicinal plants are planted in raised beds along the edge of the playground, allowing children to observe and learn about medicinal plants during their breaks (**Figure 11c**). On GZUCM Shizhen Herbal Garden and Yaowang Herbal Garden, where the planting area is more expansive, ridge planting is used to accommodate a wider range of species.

### 4.5 Designing immersive horticultural landscapes centered on natural healing principles and aesthetic education to provide multi-sensory experiences

A well-designed landscape can have a profound impact on physical and mental well-being. Rehabilitative landscapes, utilizing multisensory intervention mechanisms, can demonstrably enhance physiological, psychological, and behavioral well-being while substantially alleviating stress and fatigue (Wang and Tzortzi, 2023; Kim et al., 2024; Baik et al., 2024). Herbal gardens, by scientifically arranging plant species that exhibit both morphological diversity and aromatic characteristics, create a composite landscape ecosystem that engages visual, olfactory, and tactile senses in synergy. This design not only meets aesthetic and ornamental needs but also markedly enhances therapeutic effectiveness. Similarly, the aesthetic qualities of such landscapes make them ideal platforms for ecological and aesthetic education. Sections 3.5 and 3.6 of this study systematically summarize the aromatic active components and ornamental value of medicinal plants, providing solid scientific support for the landscape design of herbal gardens. By organically incorporating science communication and interpretive systems into landscape design, visitors are guided to perceive natural principles through aesthetic experiences, achieving the dual goals of therapeutic function and aesthetic education. In the planning and design of the GZUCM Herbal Garden, TCM culture was innovatively combined with an aesthetically pleasing herbal garden landscape, resulting in a distinctive practice base for “aesthetic education”. This project was recognized as an outstanding example of innovation in aesthetic education reform in higher education, winning the Second Prize of the 2024 Guangdong Provincial Higher Education Aesthetic Education Reform and Innovation Excellence Awards.

## 5 Limitations and future outlook

This study focused on the GZUCM Herbal Garden, systematically analyzed 1,278 species across 190 families that have been successfully conserved, and proposed applied strategies for herbal garden design and planning. Compared with the study by Gisela Mabel Paz Perafan on 106 medicinal plant species in the central mountain ranges of Colombia (Perafan and Paz, 2024) and the statistical study by Salman D. Al-Kofahi on 223 medicinal plant species in Amman, Jordan (Al-Kofahi et al., 2024), the present study covered a wider and more representative range of plant species. However, the generalizability of the conclusions is primarily constrained by factors such as regional climatic conditions and the composition of the local flora. As a result, the findings are most applicable to herbal gardens located in similar climatic zones and with comparable plant assemblages, limiting their direct global applicability. Nevertheless, the research methodology and the proposed applied strategies can serve as valuable references for the planning and design of herbal gardens in other regions. It is important to note that data collection for this study relied mainly on existing literature sources, with limited support from long-term, continuous field observations. This imposes certain limitations on systematically investigating plant growth dynamics and functional evolution. Future research aims to expand the scope by conducting comparative studies across herbal gardens in diverse climatic zones and of varying scales, while emphasizing long-term fixed-point monitoring. Such efforts will allow for a deeper exploration of the interaction mechanisms between medicinal plant growth cycles and environmental factors, ultimately providing more precise theoretical foundations for the scientific planning and rational design of herbal gardens. It is noteworthy that in 1987, Professor Honghua Xu from our university played a pivotal role in the establishment of the Chinese Herbal Garden at the UC Botanical Garden, University of California, Berkeley. This garden remains open to the public to this day (University of California Botanical Garden, 2025). Looking ahead, we aspire to disseminate the culture of traditional Chinese medicine and medicinal plants more broadly across multiple regions.

## 6 Conclusion

Medicinal plants are highly diverse, with each species possessing unique characteristics. In the planning and design of a herbal garden, it is essential to thoroughly explore the diverse attributes of these plants. Through interdisciplinary integration (ecology, TCM, psychology, etc.), the garden should be developed as a comprehensive platform that supports species conservation, educational outreach, health promotion, and cultural heritage preservation, providing the public with multi-dimensional displays of medicinal plants.

## Data Availability

The original contributions presented in the study are included in the article/**Supplementary Material**. Especially, the entire dataset is available in **Supplement Data Sheet 4**. Further inquiries can be directed to the corresponding author/s.
